# The salvage therapy in lung adenocarcinoma initially harbored susceptible *EGFR* mutation and acquired resistance occurred to the first-line gefitinib and second-line cytotoxic chemotherapy

**DOI:** 10.1186/s40360-017-0130-0

**Published:** 2017-05-10

**Authors:** Chih-Jen Yang, Jen-Yu Hung, Ming-Ju Tsai, Kuan-Li Wu, Ta-Chih Liu, Shah-Hwa Chou, Jui-Ying Lee, Jui-Sheng Hsu, Ming-Shyan Huang, Inn-Wen Chong

**Affiliations:** 1 Department of Internal Medicine, Kaohsiung Municipal Ta-Tung Hospital, Kaohsiung Medical University, Kaohsiung, Taiwan; 2 Division of Pulmonary and Critical Care Medicine, Kaohsiung Medical University Hospital, Kaohsiung Medical University, Kaohsiung, Taiwan; 3 Division of Hematology and Oncology, Department of Internal Medicine, Kaohsiung Medical University Hospital, Kaohsiung Medical University, Kaohsiung, Taiwan; 4 Faculty of Medicine, College of Medicine, Kaohsiung Medical University, No. 100, Tzyou first Road, Kaohsiung, Taiwan; 5 Graduate Institute of Medicine, College of Medicine, Kaohsiung Medical University, Kaohsiung, Taiwan; 6 Division of Chest Surgery, Department of Surgery, Kaohsiung Medical University Hospital, Kaohsiung Medical University, Kaohsiung, Taiwan; 7 Department of Medical Imaging, Kaohsiung Medical University Hospital, Kaohsiung Medical University, Kaohsiung, Taiwan

**Keywords:** Epidermal growth factor receptor, Gefitinib, Acquired resistance, Chemotherapy, Erlotinib

## Abstract

**Background:**

Epidermal growth factor receptor-tyrosine kinase inhibitors (*EGFR*-TKIs) such as gefitinib can provide better efficacy and prolonged progression free survival (PFS) than cytotoxic chemotherapy for metastatic lung non-squamous cell carcinoma harboring susceptible *EGFR* mutations when used as first-line therapy. Cytotoxic chemotherapy is regarded as being the standard therapy to overcome acquired resistance to an initial *EGFR* TKI. However, there is currently no consensus on how best to treat patients who develop resistance to both an initial *EGFR* TKI and chemotherapy.

**Methods:**

We enrolled stage IV lung adenocarcinoma patients with an *EGFR* mutation and who had developed acquired resistance to gefitinib and cytotoxic chemotherapy from two university-affiliated hospitals in Taiwan from June 2011 to December 2014. Basic demographic data, included Eastern Cooperative Oncology Group (ECOG) performance status were collected, and the response rate, progression-free survival (PFS) and overall survival (OS) were analyzed.

**Result:**

Two hundred and nine patients with mutated EGFR and who took gefitinib as the first-line therapy were identified in the study period, of whom 86 received second-line cytotoxic chemotherapy, and 60 who received third-line therapy were eligible for this study. The patients who received cytotoxic chemotherapy had a significantly higher disease control rate than those who received erlotinib (73% vs. 46%, *p* = 0.0363), however there were no significant differences in PFS (2.9 months vs. 3.1 months, *p* = 0.9049) and OS (8.9 months vs. 7.9 months, *p* = 0.4956). Platinum- or pemetrexed-based chemotherapy provided similar PFS and OS as others did. The only significant poor prognostic factors for OS were old age (≥65 years) (HR = 5.97 [2.65–13.44], *p* < 0.0001) and poor performance status (ECOG ≥2) (HR = 5.84 [2.61–13.09], *p* < 0.0001).

**Conclusion:**

Retreatment with an *EGFR* TKI is not inferior to cytotoxic chemotherapy when used as salvage therapy for patients with adenocarcinoma with an *EGFR* mutation, especially if a third-generation *EGFR* TKI is not available, or if the reason for resistance is unknown or is not related to the T790M mutation. Old age and poor ECOG score were both poor prognostic factors in the salvage therapy.

## Background

Lung cancer continues to be the leading cause of death among patients with malignant tumors worldwide. Several large scaled studies showed epidermal growth factor receptor (EGFR) - tyrosine kinase inhibitors (TKIs) were associated with a good response rate approximating 70% as well as a progression-free survival (PFS) rate of 9-13 months in patients with lung cancer harboring *EGFR* activating mutations [1–5]. However, the development of acquired resistance to the first-line *EGFR*-TKI treatment is inevitable and most of these patients needed subsequent salvage therapy [6, 7].

Because approximately half of the acquired resistance comes from T790M mutation, some new drugs were designed to conquer this resistance [8–12]. However, these new drugs were not available worldwide, included in Taiwan. In addition, not all patients could be proved to have T790M mutation because re-biopsy was usually not available [13]. Furthermore, up to 50% of the acquired resistance are not related to T790M mutation and are still unresolvable [6, 7]. In clinical practice, cytotoxic chemotherapy was considered to be the better treatment than another subsequent *EGFR*-TKI when resistance developed. In our prior report, we had demonstrated pemetrexed-based platinum chemotherapy maybe the most optimal second-line cytotoxic chemotherapy and it could prolong the PFS and overall survival (OS) [14]. Moreover, the development of acquired resistance to the second-line therapy is still inevitable. Generally speaking, cytotoxic chemotherapy is the most common choice to treat acquired resistance. Recently, Becker et al. demonstrated that retreatment with *EGFR*-TKI was an option for patients with NSCLC who were initially benefited from previous *EGFR*-TKI treatment [15]. Several small-scale studies and case reports on retreatment with the same or another *EGFR*-TKI have been published, however the results have been inconsistent [16–24]. Till now, no study was design to compare the efficacy of readministered *EGFR*-TKI or cytotoxic chemotherapy as the third-line in mutated NSCLC patients.

We therefore conduct a retrospective cohort study in two university-affiliated hospitals in Taiwan and try to explore the most optimal third-line treatment to these patients with stage IV lung adenocarcinoma initially harboring susceptible *EGFR* mutation and who finally developed acquired resistance to the front-line therapy in real world.

## Methods

### Patient identification

Patients with stage IV lung adenocarcinoma was diagnosed and treated between June 2011 and December 2014 in two Kaohsiung Medical University affiliated hospitals (Kaohsiung Medical University Hospitals and Kaohsiung Municipal Ta-Tung Hospital) in Taiwan were identified and followed until June 2015. The diagnosis of lung cancer was confirmed pathologically according to World Health Organization pathology classification, and tumor staging was made by a special committee including clinical pulmonologists, medical oncologists, chest surgeons, radiologists, pathologists and radiation oncologists according to the seventh American Joint Committee on Cancer staging system. Patients were included if they: (1) had adequate tumor specimens for *EGFR* mutation examination and had susceptible *EGFR* mutation; (2) were treated with gefitinib as the first line and subsequently received cytotoxic chemotherapy as the second-line treatment; (3) received *EGFR*-TKI or cytotoxic chemotherapy as the third-line treatment.

Baseline clinical characteristics were determined by retrospective chart review, including age at diagnosis, gender, Eastern Cooperative Oncology Group (ECOG) performance status at the beginning of the first-line gefitinib treatment and at the start of the second- and third-line treatment, smoking history and tumor histology. Mutations in the *EGFR* gene were analyzed using an *EGFR* RGQ kit (Qiagen,UK) which utilized amplification refractory mutation specific (ARMS) PCR polymerase chain reactions and Scorpion technologies for detection and/or direct sequencing as our previous report. The initial treatment response was classified based on serial imaging studies using the revised Response Evaluation Criteria in Solid Tumors (RECIST 1.1) criteria. The third-line cytotoxic chemotherapy included docetaxel, pemetrexed, vinorelbin, gemcitabine and with or without platinum derivatives (cisplatin or carboplatin).

The progression-free survival (PFS3) and overall survival (OS3) on the third-line treatment were defined as the durations from the start of the third-line treatment to the date of disease progression on imaging exam and the date of death, respectively.

The Institutional Review Board (IRB) of Kaohsiung Medical University Hospital (KMUH) approved this study (KMUHIRB-E(II)-20150162). Considering the retrospective nature of the study, we could not obtain patients consent for use of clinical data. IRB of KMUH waived the need for written informed consent from the patients. In addition, patient records were anonymous and de-identified prior to the analyses.

### Statistical analysis

Categorical variables and continuous variables were compared using the *χ* ^2^ test and the Student’s *t*-test, respectively. Survival times were estimated using the Kaplan-Meier method, with differences between the groups compared using the log-rank test. Cox regression analyses were used to determine the predicting factors for PFS3 and OS3. All statistical analyses were performed using SAS software (version 9.3 for Windows, SAS Institute Inc., Cary, NC, USA). Statistical significance was set at a two-sided *p* value of less than 0.05.

## Result

### Patient characteristics

During the study period, a total of 209 patients with stage IV lung adenocarcinoma harboring susceptible EGFR mutation who had received gefitinib as the first-line therapy were enrolled, and 86 of them had received cytotoxic chemotherapy as their second-line treatment. From these patients, 60 of them received a third-line treatment, including 29 (48%), 1 (2%), and 30 (50%) patients received erlotinib, gefitinib, and cytotoxic chemotherapy as their third-line treatment, respectively (Table 1). One patient received both erlotinib and bevacizumab and one patient received gefitinib as the third-line treatment were excluded for our subsequent analyses, because the main objective of this study was to compare the outcomes of using erlotinib alone and those of using cytotoxic chemotherapy as the third-line treatment. As summarized in Table 2, there were no significant differences in the baseline clinical characteristics between the patients receiving cytotoxic chemotherapy and those receiving erlotinib as their third-line treatment.

**Table 1:** Regimens used as the third-line treatment

Regimen	All patients	Study cohort
Erlotinib	29 (48%)^a^	28 (48%)
Gefitinib	1 (2%)	
Chemotherapy without platinum:		
Pemetrexed	1 (2%)	1 (2%)
Gemcitabine	7 (12%)	7 (12%)
Vinorelbine	7 (12%)	7 (12%)
Taxanes	5 (8%)	5 (9%)
Platinum-based doublet:		
Pemetrexed + Platinum	4 (7%)	4 (7%)
Gemcitabine + Platinum	1 (2%)	1 (2%)
Vinorelbine + Platinum	2 (3%)	2 (3%)
Taxanes + Platinum	3 (5%)	3 (5%)
Total	60	58

Data are presented as n (%)

^a^Including one patient receiving both bevacizumab and erlotinib

**Table 2:** Clinical characteristics and treatment response of the study cohort

Variables	All patients	Chemotherapy	Erlotinib	*P* value
N (%)	58	30	28	
Age (year) -mean ± SD	60.8 ± 10.8	59.2 ± 11	62.6 ± 10.4	0.2228
Age -n (%)				0.6431
<65 years old	39 (67%)	21 (70%)	18 (64%)	
≥65 years old	19 (33%)	9 (30%)	10 (36%)	
Sex-n (%)				0.1949
Female	38 (66%)	22 (73%)	16 (57%)	
Male	20 (34%)	8 (27%)	12 (43%)	
Smoking history-n (%)				0.4214
Never smoker	45 (78%)	22 (73%)	23 (82%)	
Ever smoker	13 (22%)	8 (27%)	5 (18%)	
TTF-1 staining-n (%)				0.3411
Negative	0 (0%)	0 (0%)	0 (0%)	
Positive	52 (90%)	28 (93%)	24 (86%)	
Not performed	6 (10%)	2 (7%)	4 (14%)	
EGFR gene mutation site-n (%)				0.6671
Exon18	3 (5%)	1 (3%)	2 (7%)	
Exon19	30 (52%)	16 (53%)	14 (50%)	
Exon19 + Exon21	1 (2%)	0 (0%)	1 (4%)	
Exon21	24 (41%)	13 (43%)	11 (39%)	
Metastatic sites on initial diagnosis-n (%)				0.3083
≤1	23 (40%)	10 (33%)	13 (46%)	
≥2	35 (60%)	20 (67%)	15 (54%)	
Performance status while starting gefitinib-n (%)				0.1307
ECOG ≤1	48 (83%)	27 (90%)	21 (75%)	
ECOG ≥2	10 (17%)	3 (10%)	7 (25%)	
Progression-free survival of gefitinib (month) -median (IQR)	9.4 (6.2-13.8)	9.8 (6.1-12.5)	9.1 (7.6-16.1)	0.3466
Performance status while starting the second-line treatment-n (%)				0.8848
ECOG ≤1	43 (74%)	22 (73%)	21 (75%)	
ECOG ≥2	15 (26%)	8 (27%)	7 (25%)	
Progression-free survival of the second-line treatment (month) -median (IQR)	4.1 (2.7-6.1)	3.5 (2.0-4.7)	5.0 (2.9-7.0)	0.1836
Performance status while starting the third-line treatment-n (%)				0.0606
ECOG ≤1	26 (45%)	17 (57%)	9 (32%)	
ECOG ≥2	32 (55%)	13 (43%)	19 (68%)	
Initial treatment response to the third-line treatment-n (%)				0.0842
Partial response	1 (2%)	1 (3%)	0 (0%)	
Stable disease	34 (59%)	21 (70%)	13 (46%)	
Progressive disease	23 (40%)	8 (27%)	15 (54%)	
Initial disease control rate with the third-line treatment (%)	59%	73%	46%	0.0363

### Similar outcomes while using either chemotherapy or erlotinib as the third-line treatment

**Fig. 1:**
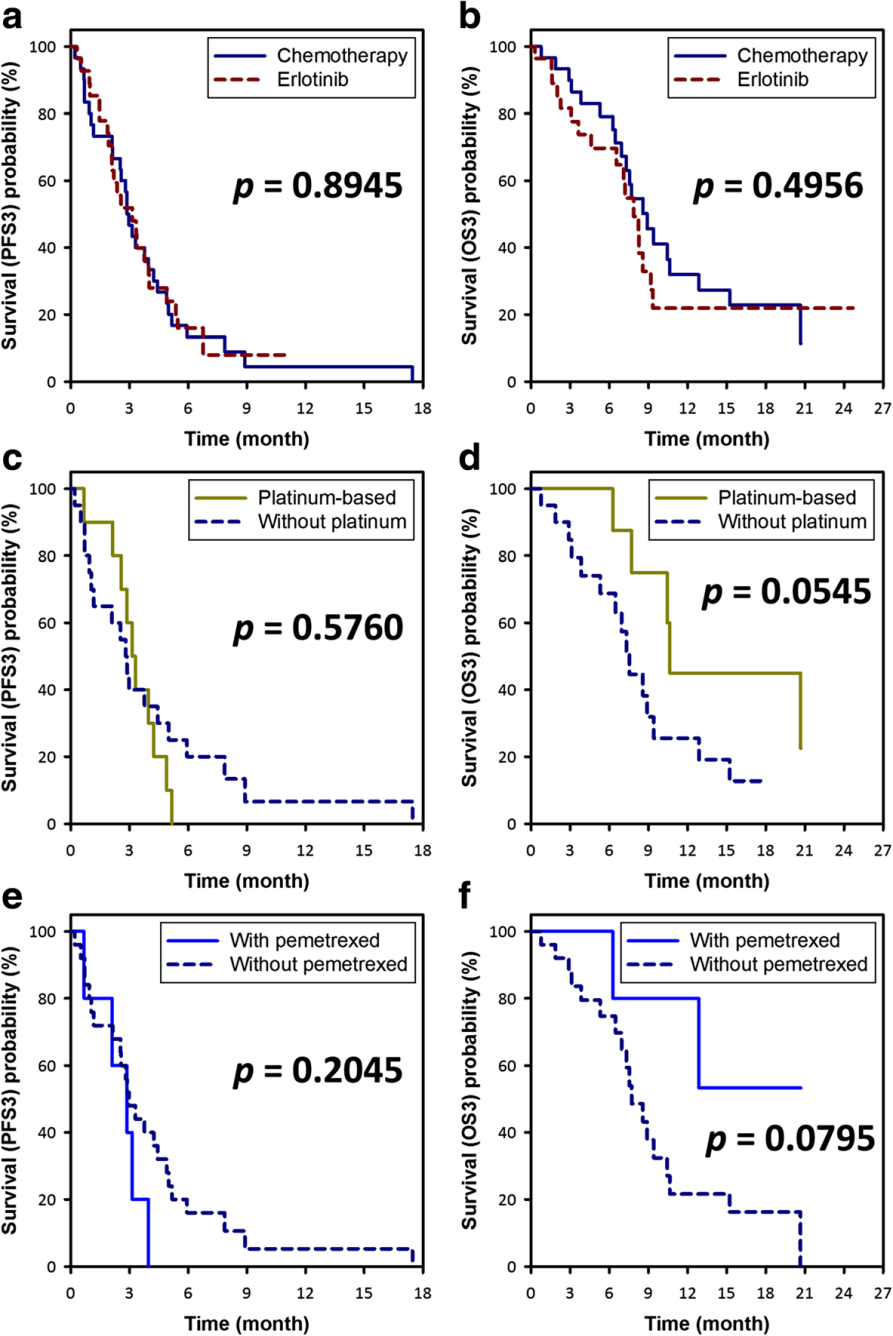
Kaplan-Meier curves of progression-free survival (PFS3; A,C,E) and overall survival (OS3; B,D,F) with the third-line treatment. **a**, **b** Analyses of the whole study cohort showed that patients receiving chemotherapy and those receiving erlotinib had similar PFS3 (MST of PFS3: 2.9 vs. 3.1 months, log-rank *p* = 0.8945) and OS3 (MST of OS3: 8.9 vs. 7.9 months, log-rank *p* = 0.4956). **c**, **d** Analyses of the patients receiving chemotherapy showed that patients receiving platinum-based doublet had a similar PFS3 and a trend for better OS3 as compared with those receiving chemotherapy without platinum (MST of PFS3: 3.2 vs. 2.8 months, log-rank *p* = 0.5760; MST of OS3: 10.6 vs. 7.5 months, log-rank *p* = 0.0545). **e**, **f** Analyses of the patients receiving chemotherapy showed that patients receiving pemetrexed had a similar PFS3 and a trend for better OS3 as compared with those receiving chemotherapy without pemetrexed (MST of PFS3: 2.9 vs. 3.0 months, log-rank *p* = 0.2045; MST of OS3: undefined vs. 7.7 months, log-rank *p* = 0.0795). Abbreviation: MST = median survival time

While patients with poorer performance status while starting the third-line treatment tended to choose erlotinib as their third-line treatment (*p* = 0.0606), patients using cytotoxic chemotherapy as the third-line treatment seemed having a significantly higher initial disease control rate than those using erlotinib (73% vs. 46%, *p* = 0.0363) (Table 2). Patients receiving chemotherapy and those receiving erlotinib had similar PFS3 (median PFS3: 2.9 vs. 3.1 months, log-rank *p* = 0.8945) and OS3 (median OS3: 8.9 vs. 7.9 months, log-rank *p* = 0.4956) (Fig. 1a, b). On multivariable Cox regression analysis controlling for sex, age, and the performance status, the use of erlotinib as the third-line treatment was not a significant predicting factor for PFS3 (HR = 0.79 [0.43–1.44], *p* = 0.4341) or OS3 (HR = 0.82 [0.41–1.64], *p* = 0.5706) (Table 4). The only significant poor prognostic factor for PFS3 was the poorer performance status (ECOG ≥2) while starting the third-line treatment (HR = 2.21 [1.20–4.07], *p* = 0.0109). The only significant poor prognostic factors for OS3 were elder age (≥65) (HR = 5.97 [2.65–13.44], *p* < 0.0001) and the poorer performance status (ECOG ≥2) while starting the third-line treatment (HR = 5.84 [2.61–13.09], *p* < 0.0001).

### Outcomes of patients using chemotherapy as the third-line treatment

We further analyzed the outcomes of patients using cytotoxic chemotherapy as their third-line treatment (Table 3). Patients receiving platinum-based doublet, as compared with those receiving chemotherapy without platinum, had a similar PFS3 (median PFS3: 3.2 vs. 2.8 months, log-rank *p* = 0.5760) and a trend for better OS3 (median OS3: 10.6 vs. 7.5 months, log-rank *p* = 0.0545) (Fig. 1c, d). Patients receiving pemetrexed, as compared with those receiving chemotherapy without pemetrexed, had a similar PFS3 (median PFS3: 2.9 vs. 3.0 months, log-rank *p* = 0.2045) and a trend for better OS3 (median OS3: undefined vs. 7.7 months, log-rank *p* = 0.0795) (Fig. 1e, f). On multivariable Cox regression analysis, the only significant poor prognostic factor for PFS3 was the poorer performance status (ECOG ≥2) while starting the third-line treatment (HR = 2.88 [1.12–7.36], *p* = 0.0278), while the use of platinum-based doublet (*p* = 0.9513) or pemetrexed-based regimen (*p* = 0.1673) was not a significant predicting factor for PFS3 (Table 4). In terms of OS3, elder age (≥65) (HR = 4.79 [1.40–16.41], *p* = 0.0126) and poorer performance status (ECOG ≥2) while starting the third-line treatment (HR = 19.78 [4.39–89.03], *p* = 0.0001) significantly predicted poorer OS3, while the use of pemetrexed-based regimen as the third-line treatment significantly predicted better OS3 (HR = 0.14 [0.02–0.89], *p* = 0.0366).

**Table 3:** Clinical characteristics and treatment response of all patients receiving cytotoxic chemotherapy as the third-line treatment

Variables	Chemotherapy without platinum	Platinum-based doublet	*P* value	Without pemetrexed	With pemetrexed	*P* value
N (%)	20	10		25	5	
Age (year) -mean ± SD	59.2 ± 12.3	59 ± 8.3	0.9665	59.8 ± 11.4	56.1 ± 9.1	0.4982
Age -n (%)			0.0910			0.1088
<65 years old	12 (60%)	9 (90%)		16 (64%)	5 (100%)	
≥65 years old	8 (40%)	1 (10%)		9 (36%)	0 (0%)	
Sex-n (%)			0.1444			0.1396
Female	13 (65%)	9 (90%)		17 (68%)	5 (100%)	
Male	7 (35%)	1 (10%)		8 (32%)	0 (0%)	
Smoking history-n (%)			0.1444			0.7119
Never smoker	13 (65%)	9 (90%)		18 (72%)	4 (80%)	
Ever smoker	7 (35%)	1 (10%)		7 (28%)	1 (20%)	
TTF-1 staining-n (%)			0.6048			0.5127
Negative	0 (0%)	0 (0%)		0 (0%)	0 (0%)	
Positive	19 (95%)	9 (90%)		23 (92%)	5 (100%)	
Not performed	1 (5%)	1 (10%)		2 (8%)	0 (0%)	
EGFR gene mutation site-n (%)			0.0225			0.1909
Exon18	0 (0%)	1 (10%)		1 (4%)	0 (0%)	
Exon19	14 (70%)	2 (20%)		15 (60%)	1 (20%)	
Exon21	6 (30%)	7 (70%)		9 (36%)	4 (80%)	
Metastatic sites on initial diagnosis-n (%)			0.5839			0.1659
≤1	6 (30%)	4 (40%)		7 (28%)	3 (60%)	
≥2	14 (70%)	6 (60%)		18 (72%)	2 (40%)	
Performance status while starting gefitinib-n (%)			0.1967			0.4142
ECOG ≤1	17 (85%)	10 (100%)		23 (92%)	4 (80%)	
ECOG ≥2	3 (15%)	0 (0%)		2 (8%)	1 (20%)	
Progression-free survival of gefitinib (month) -median (IQR)	9.3 (6.1-12.3)	10.5 (7.5-15.3)	0.5919	8.6 (6.1-11.4)	14.7 (12.5-15.4)	0.0144
Performance status while starting the second-line treatment-n (%)			0.5593			0.7119
ECOG ≤1	14 (70%)	8 (80%)		18 (72%)	4 (80%)	
ECOG ≥2	6 (30%)	2 (20%)		7 (28%)	1 (20%)	
Progression-free survival of the second-line treatment (month) -median (IQR)	4.2 (2.3-6.2)	2.5 (1.0-4.0)	0.0105	4.0 (2.4-6.0)	2.1 (1.0-2.2)	0.1359
Performance status while starting the third-line treatment-n (%)			0.2974			0.8691
ECOG ≤1	10 (50%)	7 (70%)		14 (56%)	3 (60%)	
ECOG ≥2	10 (50%)	3 (30%)		11 (44%)	2 (40%)	
Initial treatment response to the third-line treatment-n (%)			0.1513			0.0353
Partial response	0 (0%)	1 (10%)		0 (0%)	1 (20%)	
Stable disease	13 (65%)	8 (80%)		17 (68%)	4 (80%)	
Progressive disease	7 (35%)	1 (10%)		8 (32%)	0 (0%)	
Initial disease control rate with the third-line treatment (%)	65%	90%	0.1444	68%	100%	0.1396

**Table 4:** Cox regression analyses for the factors predicting progression-free survival (PFS3) and overall survival (OS3) with the third-line treatment

Clinical features	Progression-free survival with the third-line treatment (PFS3)		Overall survival with the third-line treatment (OS3)	
	Univariate analysis	Multivariable analysis	Univariate analysis	Multivariable analysis
All study cohort				
Sex (male vs. female)	0.80 [0.44 - 1.45]	0.94 [0.50 - 1.79]	0.61 [0.29 - 1.28]	0.70 [0.32 - 1.51]
Age (≥65 vs. <65 years old)	0.80 [0.44 - 1.46]	0.78 [0.43 - 1.42]	3.09 [1.52 - 6.28]	5.97 [2.65 - 13.44]
Performance status while starting the third-line treatment (ECOG ≥2 vs. ≤1)	2.08 [1.18 - 3.66]	2.21 [1.20 - 4.07]	3.30 [1.64 - 6.64]	5.84 [2.61 - 13.09]
The third-line treatment (erlotinib vs. cytotoxic chemotherapy)	0.96 [0.55 - 1.68]	0.79 [0.43 - 1.44]	1.25 [0.65 - 2.40]	0.82 [0.41 - 1.64]
Patients receiving cytotoxic chemotherapy as the third-line treatment				
Sex (male vs. female)	1.01 [0.44 - 2.34]	1.57 [0.51 - 4.81]	0.93 [0.33 - 2.59]	1.21 [0.35 - 4.19]
Age (≥65 vs. <65 years old)	0.57 [0.24 - 1.36]	0.62 [0.22 - 1.71]	3.10 [1.11 - 8.69]	4.79 [1.40 - 16.41]
Performance status while starting the third-line treatment (ECOG ≥2 vs. ≤1)	2.00 [0.93 - 4.31]	2.88 [1.12 - 7.36]	6.62 [2.35 - 18.64]	19.78 [4.39 - 89.03]
The third-line treatment (platinum-based doublet vs. chemotherapy without platinum)	1.26 [0.56 - 2.84]	0.97 [0.38 - 2.46]	0.35 [0.12 - 1.07]	1.12 [0.31 - 4.07]
The third-line treatment (with pemetrexed vs. without pemetrexed)	1.91 [0.69 - 5.27]	2.34 [0.70 - 7.84]	0.29 [0.07 - 1.26]	0.14 [0.02 - 0.89]

## Discussion

Our study was designed to determine the treatment strategies in patients who initially harbored *EGFR* mutation and developed acquired resistance to the first-line Gefitinib and second-line cytotoxic chemotherapy. We found that re-treated with *EGFR*-TKIs were not inferior to traditional cytotoxic chemotherapy if they were selected as the third-line therapy in initial *EGFR*-mutated adenocarcinoma patients. In addition, platinum doublet chemotherapy is not superior to non-platinum-based chemotherapy. Finally, no statistic profit in PFS was observed between different cytotoxic chemotherapeutic agents.

Gefitinib had been permitted by NIH bureau in Taiwan to treat patients with lung adenocarcinoma with susceptible *EGFR* mutation as the first-line therapy based on several phase III studies since June 2011 [1–4]. Despite gefitinib showed good efficacy and longer PFS than cytotoxic chemotherapy, acquired resistance to *EGFR*-TKI treatment always occurred, and these patients needed subsequent cytotoxic chemotherapy as the second-line therapy. Pemetrexed based platinum chemotherapy was considered to be a promising therapy in our previous report. [14] However, the standard therapy of the third-line therapy in such patients with initially mutated *EGFR* was still uncertain. Prior second cytotoxic chemotherapy always resulted in poorer performance status and adverse drug reaction of the cytotoxic chemotherapy also causes patients to be afraid of receiving further third-line cytotoxic chemotherapy. Some studies showed lung cancer cell lines regained susceptibility to *EGFR*-TKI after resting for a period, and this was called *EGFR*-TKI holiday [16, 25]. The TKI-holiday theory was proposed by Becker et al., who showed a high response rate (36%) and disease control rate (86%) in patients who received a second *EGFR*-TKI after a median interval of about 9.5 (3–36) months [15]. They concluded that retreatment with *EGFR*-TKI was an option for patients with NSCLC who initially benefitted from previous *EGFR*-TKI treatment and then experienced recurrence after standard cytotoxic chemotherapy. Unlike traditional cytotoxic chemotherapy, *EGFR*-TKIs induced minimal hematological or non-hematological adverse drugs reaction and were considered to be well-tolerated drugs, especially for the elderly and patients with poorer performance status. Therefore, some physicians proposed that re-administration of *1* ^*st*^ *or 2* ^*nd*^ generation *EGFR* TKI to conquer acquire resistance in patients with lung non-squamous cell carcinoma initially harboring *EGFR* mutation. Since retreatment with an *EGFR*-TKI may have a good disease control rate, several case reports and case series have been published [16, 17, 20–24, 26]. Our previous report showed female ever smoker had a poorer prognosis in patients who were retreated with *EGFR*-TKI in Taiwan [27]. Furthermore, the responses to the re-administered *EGFR*-TKI as the third-line therapy in initially mutated *EGFR* patients have been inconsistent [16–24] and cytotoxic chemotherapy is still the most common salvage therapy to those having acquired resistance to second-line chemotherapy. The current cohort study indicated that *EGFR*-TKI could provide similar efficacy as traditional cytotoxic chemotherapy does.

We had proposed that pemetrexed-based platinum chemotherapy may conquer acquired resistance to the first-line *EGFR*-TKI and it was regarded as the most optimal choice for second-line therapy [14]. The median PFS was as high as 6.4 months and it was significantly longer than other platinum-based chemotherapy such as gemcitabine, vinorelbine, docetaxel. However, efficacy of cytotoxic chemotherapy is still uncertain. In all NSCLC patients, included initially *EGFR* mutated or non-mutated patients, Sun et al. reported that pemetrexed is the optimal drug with good efficacy and a tolerable toxicity if it was used as the third-line therapy, the median PFS was 2.83 months with 22% response rate [28]. Chang et al. documented a similar report that when pemetrexed was as the third- or 4^th^ –line, it provided PFS of 3.2 months, OS of 11.6 months, and the response rate of 16.3% [29]. Chen et al. showed the response rate was 10% and PFS was 2.6 months when using pemetrexed as third-line treatment, and the median survivals were 13.4 months for pemetrexed in third-line salvage therapy [30]. However, the role of pemetrexed as the third- or later-line therapy in *EGFR* mutated patients is still challenging.

In *EGFR* mutated patients, Shukuya et al. firstly showed a subgroup analysis of NSCLC patients who initially harbored *EGFR* mutation, and received single agent chemotherapy with docetaxel or pemetrexed as the 3^rd^ line therapy [31]. The response rate was 17.6% and PFS was 113 days. In our report, pemetrexed-based therapy could not show better PFS, but it was observed to contribute to a higher OS than based on based on our multivariate Cox regression model analysis though the sample size is relatively small.

There were several limitations in this study. First, our study is a retrospective study and sample size is too small. However, this is the first cohort study in the mutated adenocarcinoma patients and we demonstrated the real world data in Taiwan. Second, no re-biopsy specimens were collected and the accurate molecular mechanisms are unknown. Third, erlotinib was the re-administered *EGFR*-TKI in this study based on the Taiwan NIH policy, which covers erlotinib as the 3^rd^ line treatment for NSCLC when patients with EGFR mutations exhibit resistance to initial treatment with gefitinib and cytotoxic chemotherapy. To date, third-generation *EGFR* TKIs such as osimertinib (AZD 9291) which has been approved to overcome the T790M mutation is not permitted in Taiwan NIH and in many countries.

## Conclusion

In conclusion, we pointed out that re-administration of *EGFR*-TKI and cytotoxic chemotherapy shared the similar clinical efficacy in third-line therapy in patients who had lung adenocarcinoma initially harbored susceptible *EGFR* mutation, if the 3^rd^ *EGFR* TKI is not available, unknown resistance mechanism or not related to T790M mutation. All cytotoxic chemotherapy had similar outcome though pemetrexed seems to have better OS. Large-scaled randomized controlled trial is necessary to confirm our findings.

## Abbreviations

EGFR: Epidermal growth factor receptor
OS: Overall survival
PFS: Progression free survival
TKIs: Tyrosine kinase inhibitors
